# Supplementary material for: Twisted oxide lateral homostructures with conjunction tunability
Source: Nat Commun. 2022 May 10;13:2565. doi: 10.1038/s41467-022-30321-8 (PMC9090740; doi:10.1038/s41467-022-30321-8)
Supplement: Supplementary file 1 — Supplementary Information [file 41467_2022_30321_MOESM1_ESM.pdf]

Supplementary Information

Twisted oxide lateral homostructures with conjunction tunability

Ping-Chun Wu[1†], Chia-Chun Wei[1†], Qilan Zhong[2†], Sheng-Zhu Ho[1], Yi-De Liou[1], Yu-Chen Liu[1], Chun-Chien Chiu[1], Wen-Yen Tzeng[3], Kuo-En Chang[1], Yao-Wen Chang[1], Junding Zheng[2], Chun-Fu Chang[4], Chien-Ming Tu[3], Tse-Ming Chen[1], Chih-Wei Luo[3,5], Rong Huang[2], Chun-Gang Duan[2], Yi-Chun Chen[1], Chang-Yang Kuo[3,5] and Jan-Chi Yang[1,6*]

[1] *Department of Physics, National Cheng Kung University, Tainan, 70101, Taiwan*
[2] *Key Laboratory of Polar Materials and Devices (MOE) and Department of Electronics, East China Normal University, Shanghai, 200241, China*
[3] *Department of Electrophysics, National Yang Ming Chiao Tung University, Hsinchu, 30010, Taiwan*
[4] *Max-Planck Institute for Chemical Physics of Solids, Dresden, 01187, Germany*
[5] *National Synchrotron Radiation Research Center, Hsinchu, 30076, Taiwan*
[6] *Center for Quantum Frontiers of Research & Technology (QFort), National Cheng Kung University, Tainan, 70101, Taiwan*

*e-mail: janchiyang@phys.ncku.edu.tw

Supplementary Note 1. X-ray diffraction studies of twisted BFO.

The epitaxial relationship of the BFO lateral homostructures have been revealed by X-ray diffraction (XRD). Using the X-ray spot that covers both as-grown and freestanding regions, the out-of-plane XRD exhibits pure (110)-oriented BFO peaks without any secondary phase, indicating the same crystalline orientation along out-of-plane of (110) STO (Supplementary Figure 1a). To further confirm the twist angle relative to the two areas, in-plane φ-scan has been adopted. The reflection of BFO {221} planes contributed from BFO_{AS} and BFO_{FS} are detected every 180º, respectively, indicating the 2-fold symmetry of (110)-oriented BFO, while the φ difference shows the twist angle (~85°) between the as-grown and freestanding BFO, as illustrated in Supplementary Figure 1b.

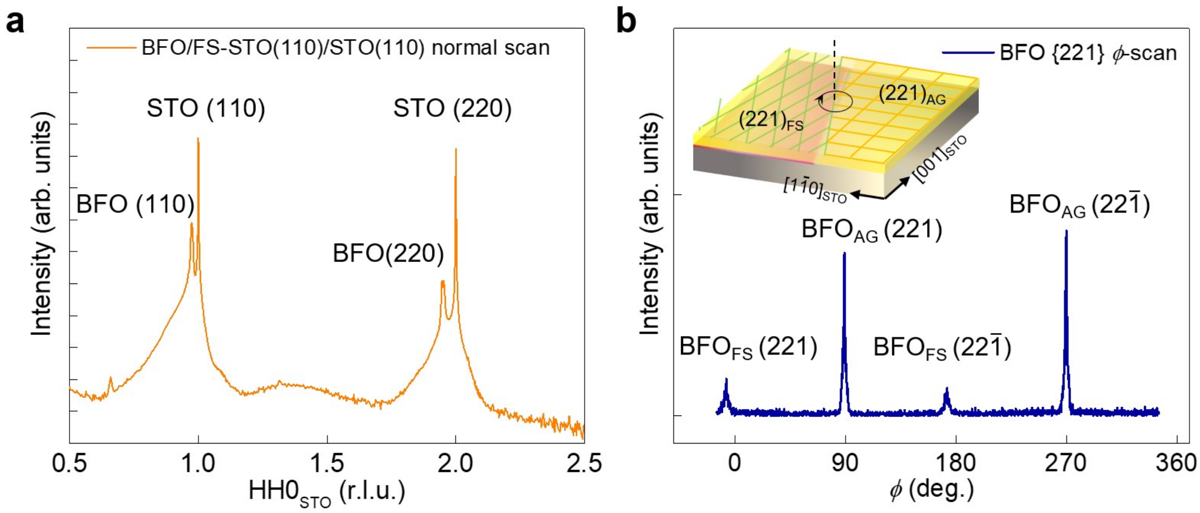

Supplementary Figure 1. X-ray diffraction (XRD) of the twisted lateral BFO homostructure. a XRD normal scan of twisted BFO homostructure on (110)-oriented STO substrate indicates pure (110)-oriented BFO peaks without any secondary phase. The reciprocal lattice unit (r.l.u) is normalized to the STO substrate (1 r.l.u. = $2\pi/a_{STO}$, a_{STO} = 3.905 Å). **b** XRD in-plane φ-scan of STO {221} and BFO {221}. The detection of 4 peaks in STO {221} planes indicates the contributions from both freestanding and single crystal STO substrate areas. In the present sample, the twist angle between single crystal STO substrate and freestanding STO is ~85º.

Supplementary Note 2. Density-functional calculation of the binding energy.

The correlation between the interlayer distance and the binding energy is established by density-functional theory (DFT) calculation. The DFT calculations are performed using the accurate full-potential projector augmented wave (PAW) method, as implemented in the Vienna ab initio simulation package (VASP). The exchange-correlation potential is treated in Perdew-Burke-Ernzerhof (PBE) form of the generalized gradient approximation (GGA) with a kinetic-energy cutoff of 500 eV. Monkhorst-Pack k-point grids (5×5×1) are adopted in calculation. The energy tolerance is 1.0×10^{-5} eV/atom. Hellmann-Feynman forces on each atom are less than 1 meV/Å in ground state. The Coulomb interaction parameter U is chosen to be 2 eV for the Fe atom.

Supplementary Figure 2 shows the calculation results on the binding energy evolution with interlayer distance of STO-STO, STO-BFO van der Waals bonding and STO-BFO ionic bonding. Interlayer distance is marked by black arrows in the schematic diagram of atomic structure. Bonding energy in ground state are -0.0482 eV, -0.1786 eV and -2.04 eV for STO-STO, STO-BFO van der Waals bonding and STO-BFO ionic bonding. It shows that STO-BFO ionic bonding energy is much higher than van der Waals bonding energy. STO-BFO van der Waals bonding is stronger than it is in STO-STO.

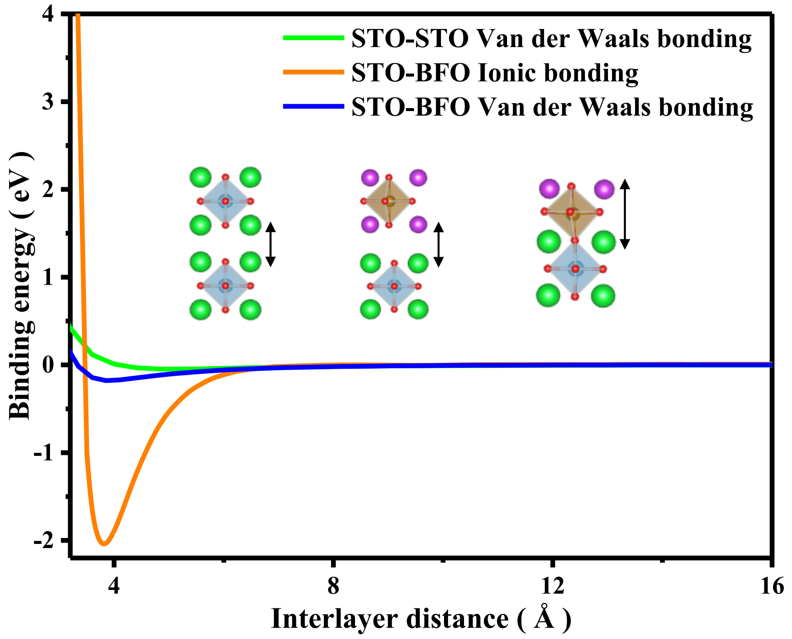

Supplementary Figure 2. Binding energy evolution with interlayer distance of STO-STO, STO-BFO van der Waals bonding and STO-BFO ionic bonding.

Supplementary Note 3. Controllable twist φ angle of the lateral homostructures.

The morphology and ferroelectric domain patterns of lateral epitaxial BFO thin film grown on twisted (110)-STO template with various twisted angles φ were characterized by PFM. The angular-dependent demonstration for small, medium, and large twist angles (5°, 45°, and 90°) are shown in Supplementary Figure 3a, 3b, and 3c, respectively. From these topography images, the twist angle between as-grown and freestanding regions can be distinguished via the [001] BFO stripes. Experimentally, with the applications of both elastic and electrostatic boundary conditions, only two of the eight possible polarization directions with upward component was allowed to be detected. In this manner, two structural variants are retained, whereby the twin-domain of BFO can thus be observed on the freestanding STO region. From previous study, the adoption of miscut STO substrate, which breaks the ferroelectric equivalency, leads to the formation of single domain BFO grown on single crystal STO substrate. Combined with previous XRD study, these results indicate that ionic bonds are formed between the freestanding STO and the BFO_{FS}, rather than weak van der Waals bonds. The corresponding domain structures have been further revealed by PFM. Supplementary Figure 3d, 3e, and 3f (in-plane PFM phase images) show the single-domain and twin-domain features of BFO grown on pristine STO and FS-STO, respectively, with different twist angles. Interestingly, in lateral homostructures with medium and large twist angles, a zigzag domain pattern with 71° domain walls emerges near the boundary of the BFO single domain side. Furthermore, the corresponding out-of-plane PFM phase exhibits uniform upward polarization on both sides of each later homostructure (Supplementary Figure 3g, 3h, and 3i), regardless of the twist angles. The observations suggest that the rotation of ferroelectric polarization toward boundaries is to stabilize the uncompensated charges at the interface between BFO_{FS} and BFO_{AG} grown on freestanding STO and substrate, respectively. In contrast, for the sample with a small twist angle, the electrostatic energy on both sides is equivalent. As a result, the single domain feature is observed at the boundary of the lateral homostructure with a small twist angle.

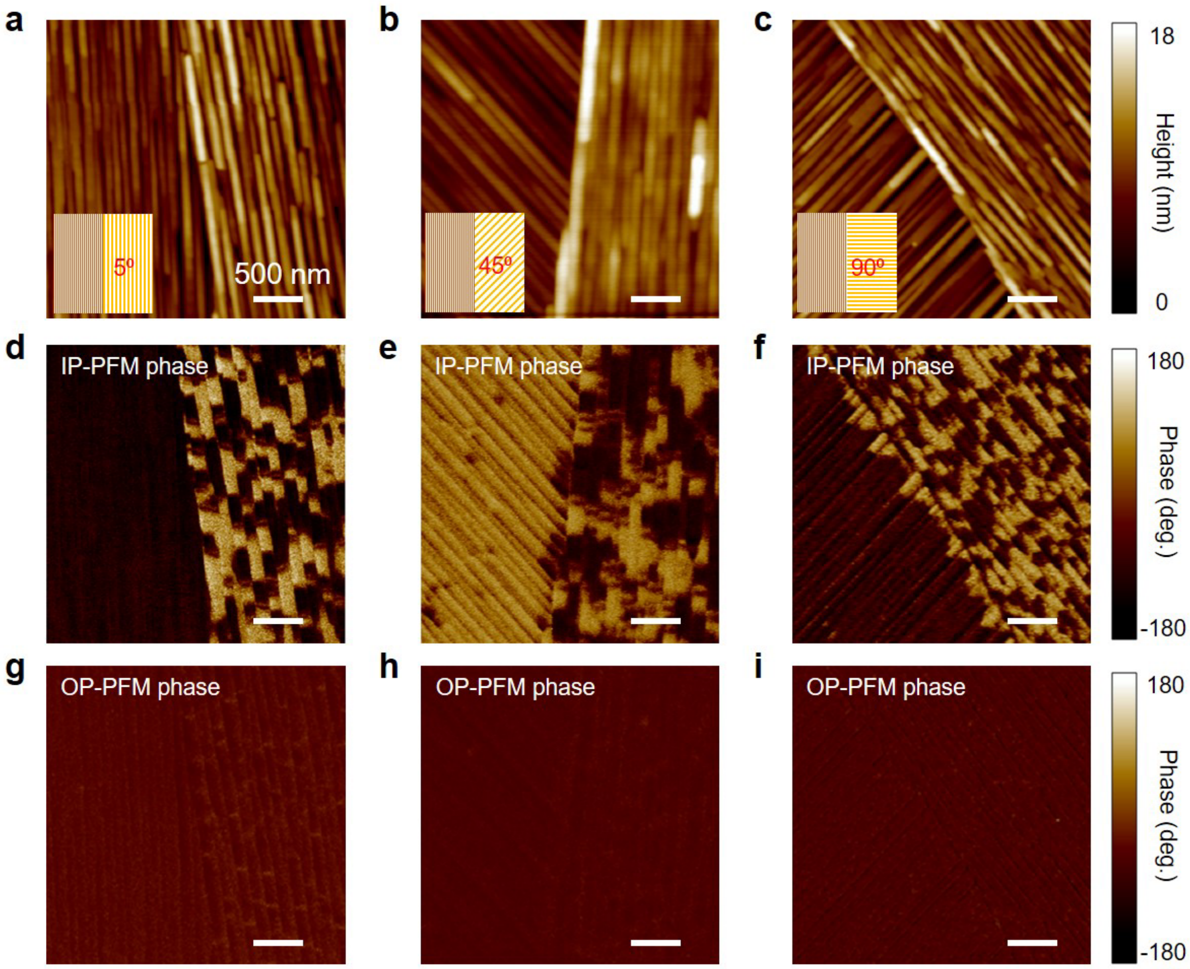

Supplementary Figure 3. Topography and domain structures of lateral homostructures with different twist angles. a, **b** and **c** Topography images of lateral homostructures with the twist angles of 5˚, 45˚, and 90˚, respectively. **d**, **e** and **f** In-plane PFM phase images for lateral homostructures with twist angles of 5˚, 45˚, and 90˚, respectively. **g**, **h** and **i** Out-of-plane PFM phase images for lateral homostructures with twist angles of 5˚, 45˚, and 90˚, respectively. The scale bars refer to 500 nm.

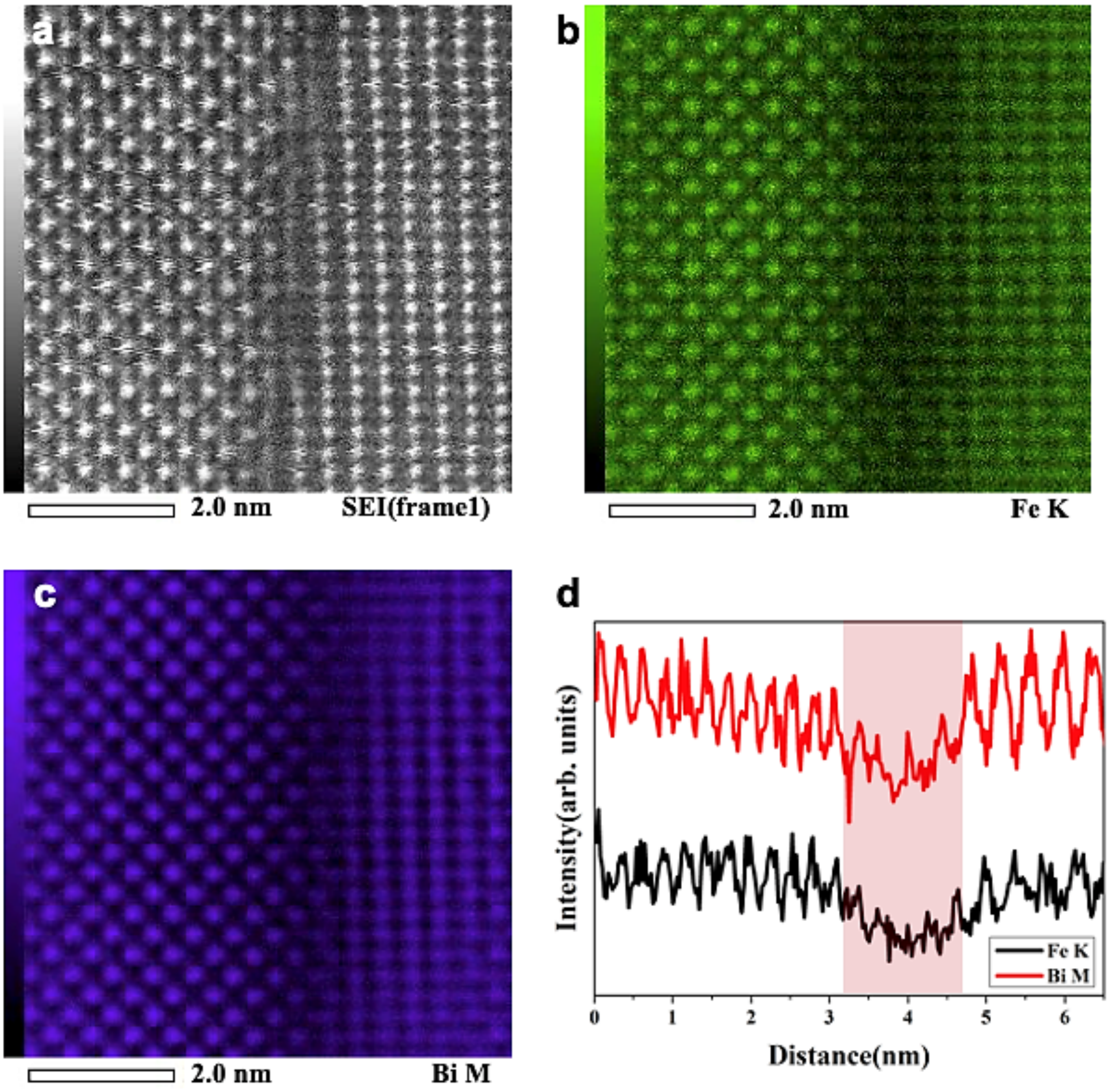

Supplementary Figure 4. Atomic resolved HAADF-STEM image and corresponding EDS mapping of 90° twisted BFO lateral homostructure. a HAADF-STEM image of the interface region in the BFO_{AG}/BFO_{FS}. **b** and **c** Atomic resolved Fe and Bi EDS map. **d** Line profiles of Fe and Bi extracted from the mapping.

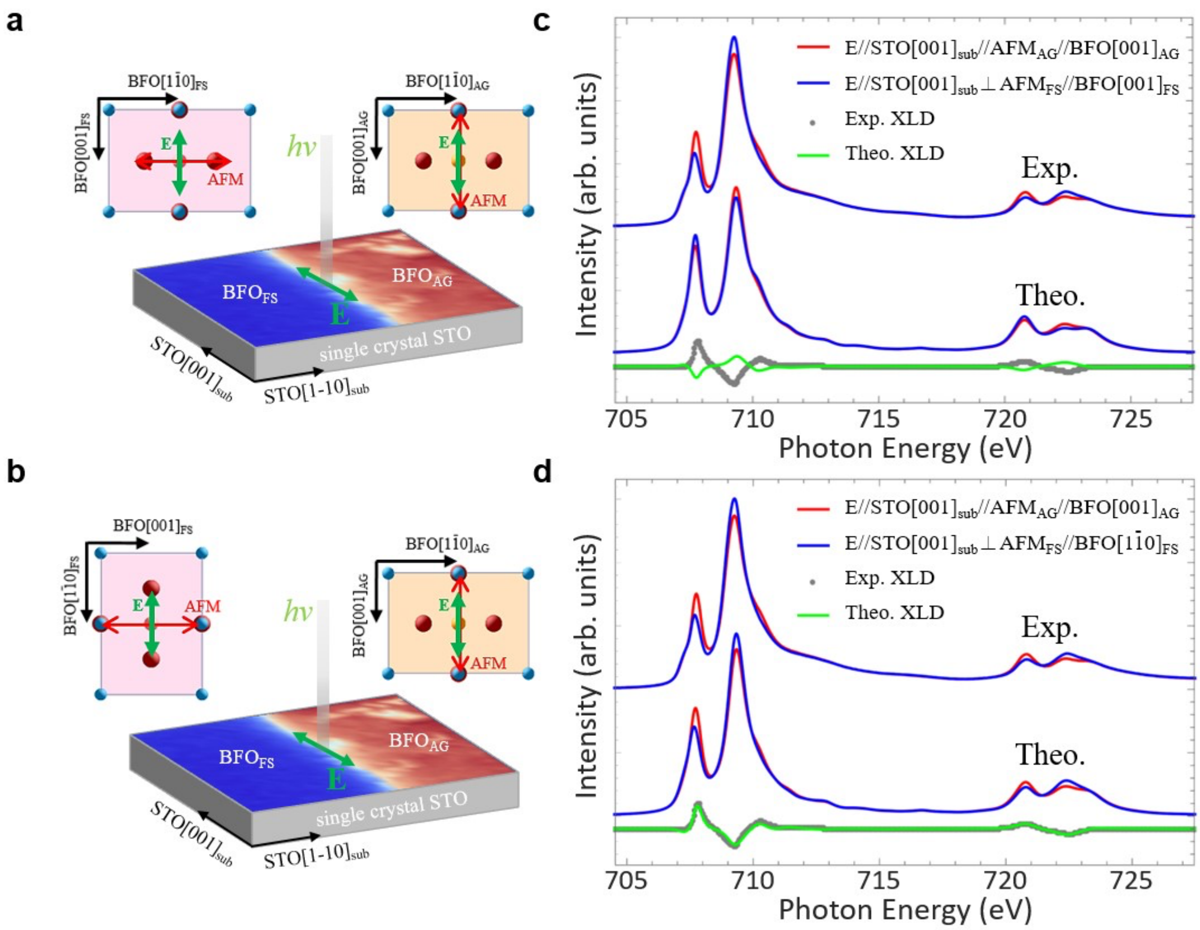

Supplementary Figure 5. X-ray absorption spectra, linear dichroism spectra, and the simulation of Fe $L_{2,3}$-edge of BFO_{AG} and BFO_{FS} with $E//STO[001]_{sub}$. a,b Schematics of simulation considering (**a**) the 90° rotation of antiferromagnetic axis from BFO_{AG} to BFO_{FS} and (**b**) the 90° rotation of both antiferromagnetic axis and [1$\bar{1}$0] crystallography axis from BFO_{AG} to BFO_{FS}. The corresponding simulated results are shown in **c** and **d**, respectively. The red and blue lines in (**c**) and (**d**) represent the absorption spectra of BFO_{AG} and BFO_{FS}, while the green lines and gray dots show the experimental and simulated dichroism spectra, respectively.

Supplementary Note 4. X-ray diffraction studies of BFO polymorphs grown on twisted template.

To reveal the conjunction tunability of polymorphs made of the same materials, freestanding STO thin film was transferred onto LAO substrate, followed by the growth of BFO thin film. Due to the lattice mismatch, BFO grown on FS-STO develops a rhombohedral-like structure (R-BFO), while BFO grown on LAO substrate progresses a tetragonal-like structure (T-BFO). Supplementary Figure 6a shows the XRD normal scan of BFO on the STO/LAO twisted template. The featured peaks of R-, T-BFO, FS-STO and LAO substrate are clearly observed without any other impurity phases. The XRD in-plane φ-scans along {103} planes of R-BFO, T-BFO, STO, and LAO indicate the well-aligned epitaxial relationship of R-BFO/FS-STO and T-BFO/LAO, respectively (Supplementary Figure 6b). Furthermore, the RSM around {113} plane of LAO revealed the single domain feature of T-BFO and the T-BFO is fully strained by LAO substrate (Supplementary Figure 6c). The RSM around {113} plane of STO also indicates a strained R-BFO phase on FS-STO (Supplementary Figure 6d).

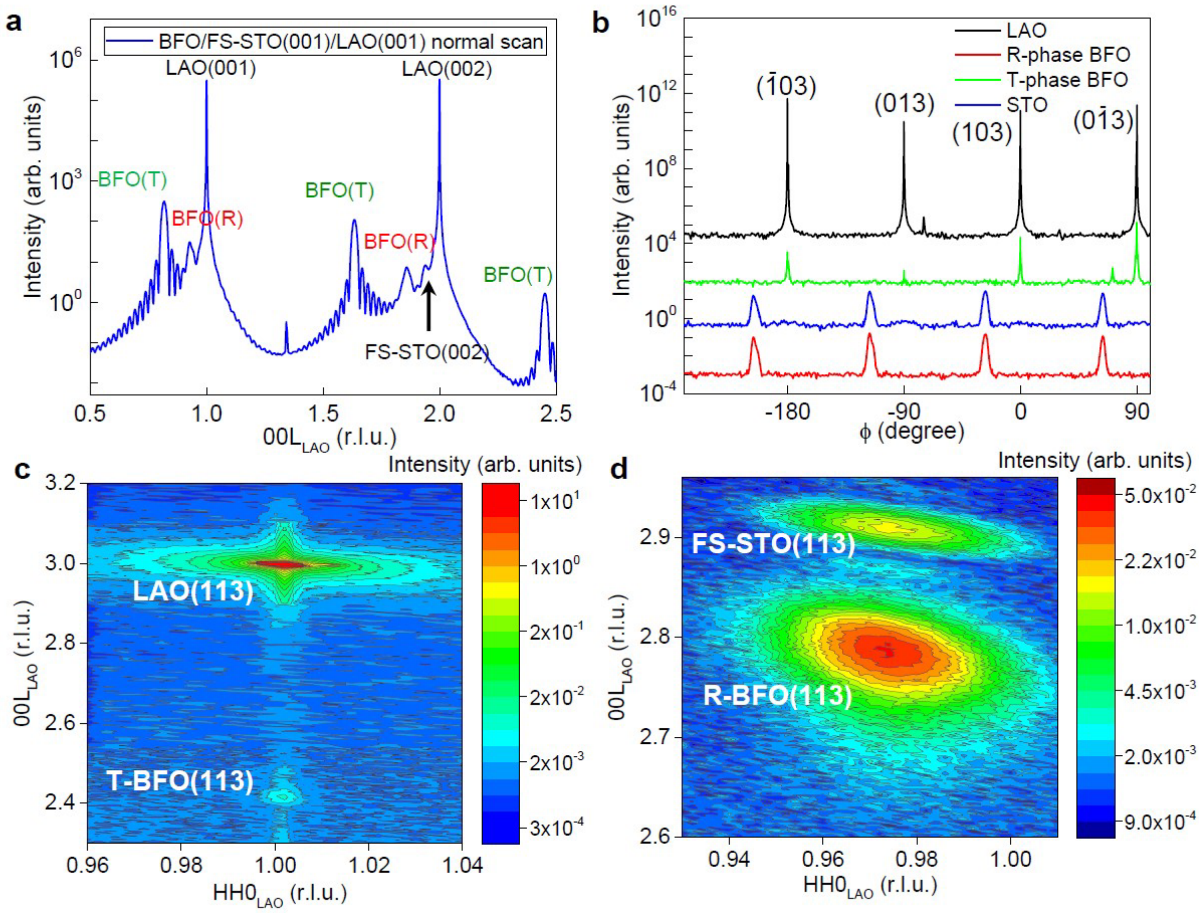

Supplementary Figure 6. X-ray diffraction of BFO grown on STO/LAO twisted template. **a** XRD normal scan along LAO (001). The characteristic peaks of R-BFO, T-BFO, FS-STO, LAO are labeled accordingly in the figure. **b** The XRD in-plane φ-scans along {103} planes of R-BFO, T-BFO, STO, and LAO indicate the well-aligned epitaxial relationship of R-BFO/FS-STO and T-BFO/LAO, respectively. **c** The RSM around {113} plane of LAO revealed the single domain feature of T-BFO and the T-BFO is fully strained by LAO substrate. **d** The RSM around {113} plane of STO also indicates a strained R-BFO phase on FS-STO. The reciprocal lattice unit (r.l.u) is normalized to the LAO substrate (1 r.l.u. = $2\pi/a_{LAO}$, a_{LAO} = 3.787 Å).

Supplementary Note 5. TEM observation of homostructure composed of BFO polymorphs.

Transmission electron microscopy is used to reveal the lateral homostructure composed of T-BFO and R-BFO. The T-BFO / R-BFO interface can be seen clearly, as marked with a yellow dashed line in Supplementary Figure 7a along the LAO [010] direction. The angle between the T/R interface and the surface of LAO substrate is about 30 degrees. The thickness of R-BFO is larger than that of T-BFO, indicating a relatively faster growth of R-BFO on the FS-STO. The HRTEM image (Supplementary Figure 7b) shows the clear and sharp interfaces including T-BFO/LAO, R-BFO/FS-STO and T-BFO/R-BFO. The heteroepitaxial relationships can be determined from the HRTEM image and the corresponding SAED patterns shown in Supplementary Figure 7c, 7d and 7e, respectively, consistent with the XRD results.

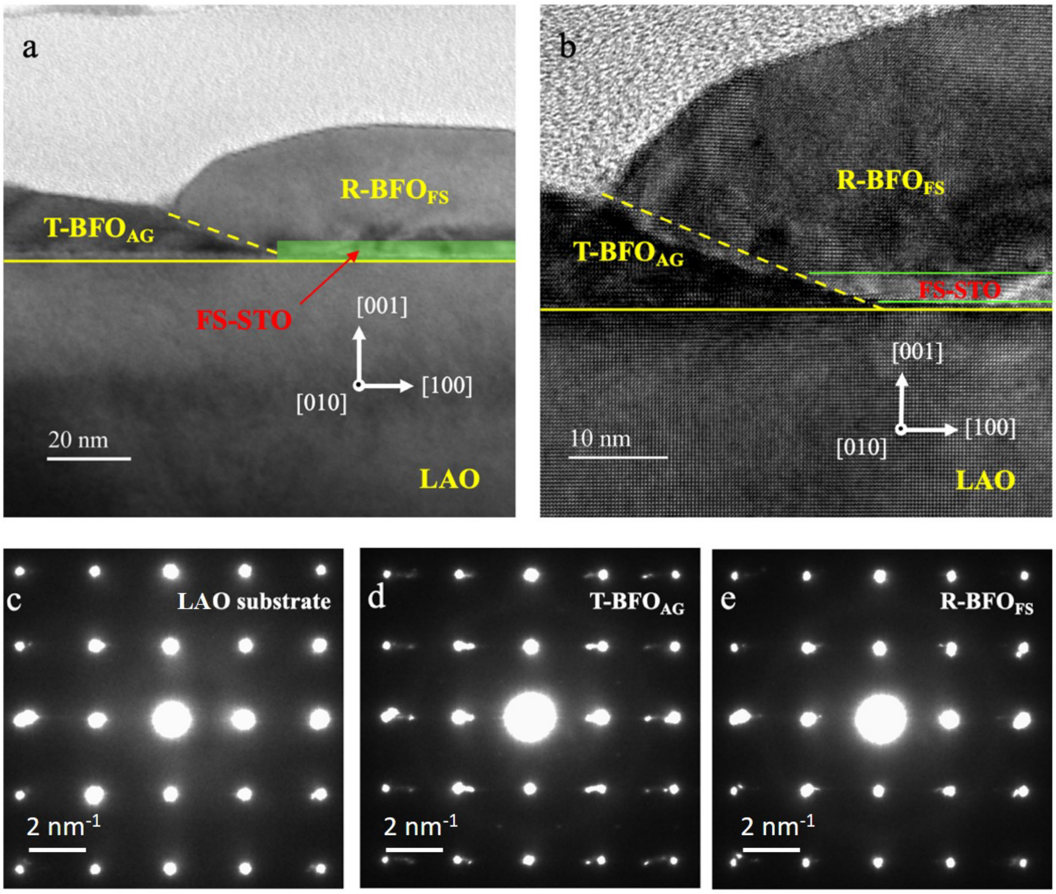

Supplementary Figure 7. Microstructure characterization of the polymorphs BFO lateral homostructure. a Typical cross-sectional TEM image taking along [010] direction of LAO substrate. **b** HRTEM images of the interface area along the [010] direction of LAO substrate. **c**, **d** and **e** SAED patterns of LAO, T-BFO and R-BFO, respectively.

Supplementary Note 6. Second harmonic generation of twisted BFO lateral homostructure.

The nonlinear optical properties of the 90° twisted BFO homostructures were studied via second harmonic generation (SHG) techniques with confocal microscopy mapping. Supplementary Figure 8a and its insets show the schematic illustration of the experiment setup, the spectra of the excitation laser beam and the typical SHG of BFO films. By the polar plots of SHG intensity (p-polarized output) in Supplementary Figure 8b and 8c, we found that the polarity of SHG intensity from BFO_{AG} and BFO_{FS} are perpendicular due to the twist angle of 90° between the as-grown BFO_{AG} and the freestanding BFO_{FS}. Supplementary Figure 8d further shows the SHG mapping across the boundary between BFO_{AG} and BFO_{FS} obtained by the confocal microscopy with a p-polarized excitation laser beam. After the 800 nm excitation beam filtered out, we clearly observed a 20 % enhancement of SHG intensity along the BFO_{AG}/BFO_{FS} boundary in the SHG mapping (see Supplementary Figure 8d) and the spectra (see Supplementary Figure 8e-g). Moreover, the SHG intensity as a function of excitation polarization angles ϕ in region 2 (BFO_{AG}/BFO_{FS} boundary) is the same as that in region 1 (BFO_{AG}). Therefore, the enhancement of SHG intensity could be attributed to the band bending or carrier accumulation due to polarization discontinuity at the twisted interface, which leads to the increase of nonlinear optical coefficient at BFO_{AG}/BFO_{FS} boundary[1,2]. These SHG results demonstrate that the polarization of SHG rotated as the BFO twisted, and an enhancement of SHG intensity at the BFO_{AG}/BFO_{FS} homojunction boundary.

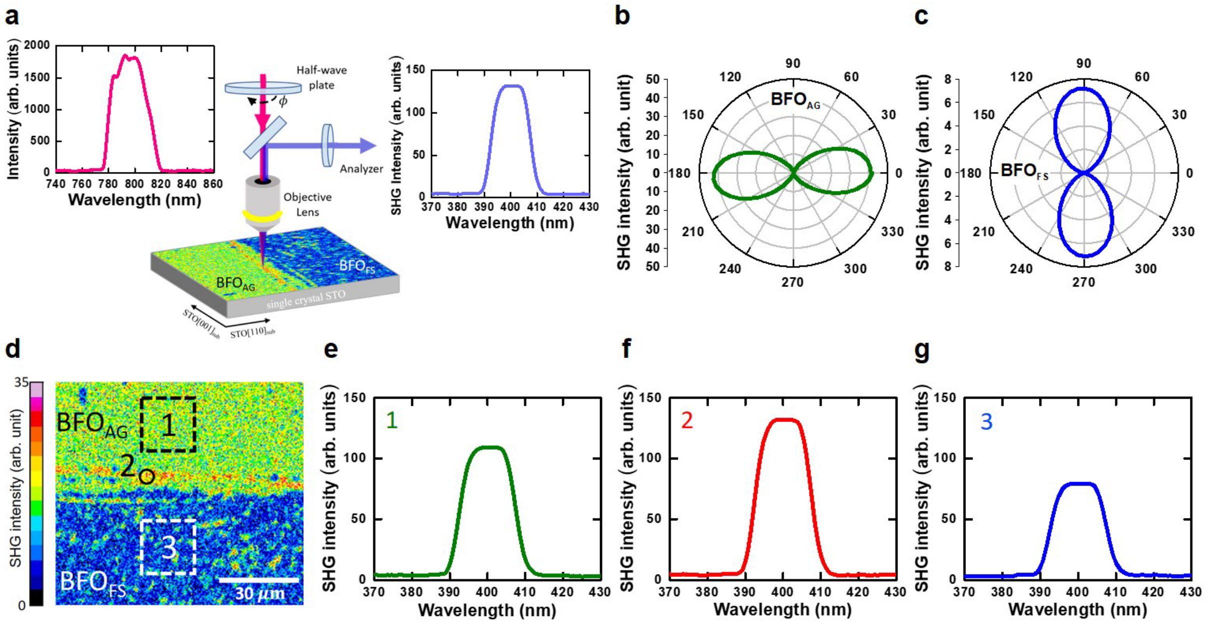

Supplementary Figure 8. Second harmonic generation and confocal microscopy mapping of 90° twisted (110)-oriented BFO lateral homostructure. a Schematic illustration of the SHG spectrograph and microscopy setup. The insets show the spectra of the excitation laser beam and the typical SHG as represented by the red and blue curves, respectively. P-polarized SHG intensity of **b** BFO_{AG} and **c** BFO_{FS} as a function of incident polarization angles ϕ manipulated by rotating the half-wave plate in (**a**). **d** The SHG intensity mapping across the boundary between BFO_{AG} and BFO_{FS}. **e-g** The SHG spectra of the BFO_{AG}, BFO_{AG}/BFO_{FS} boundary, and BFO_{FS} as marked in (**d**).

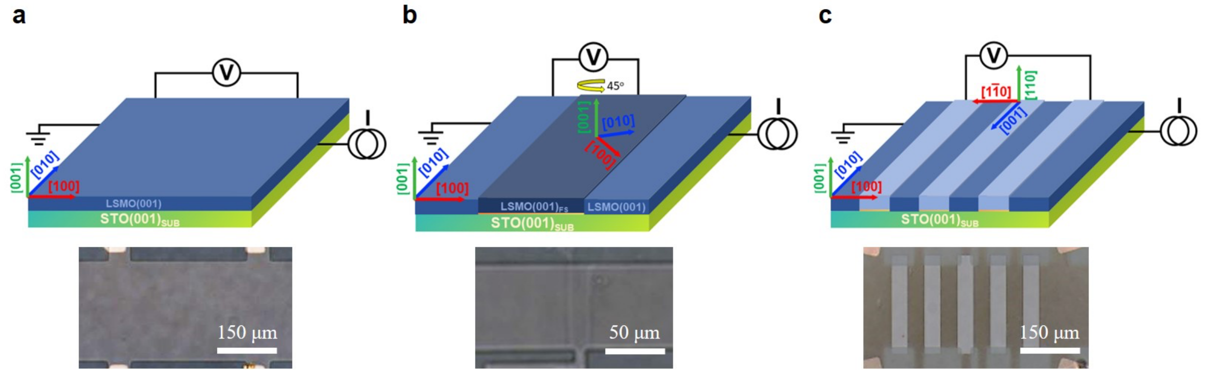

Supplementary Figure 9. Magnetotransport measurements on LSMO lateral homostructures. Device architectures of **a** pure LSMO, **b** (001)x(001)$_{45}$ LSMO, and **c** (001)x(110) LSMO samples. The top panels show the measurement Hall bar geometry, while the optical microscope images are shown in the bottom panels. The STO substrate is highlighted in green, FS-STO in yellow, and LSMO in light and dark blue. The external magnetic field is applied parallel [001] direction of STO substrates.

Supplementary Note 7. Conjunction tunability.

To demonstrate the versatile tunability based on our method, a similar approach has been adopted to fabricate (001)-oriented BFO grown on twisted STO (001) template (Supplementary Figure 10a-c). Besides, such an approach is also capable of producing lateral conjunctions with different out-of-plane crystalline directions (Supplementary Figure 10d-f). Here, freestanding (110) STO was transferred onto (100) STO substrate, followed by the deposition of BFO thin films. As shown in Supplementary Figure 10a-i, the twist angles could be easily identified via the surface topography, given that the stripes on the surface are correlated to specific crystalline directions. These results indicate that such an approach offers a universal template to the synthesis of lateral homostructures and homojunctions.

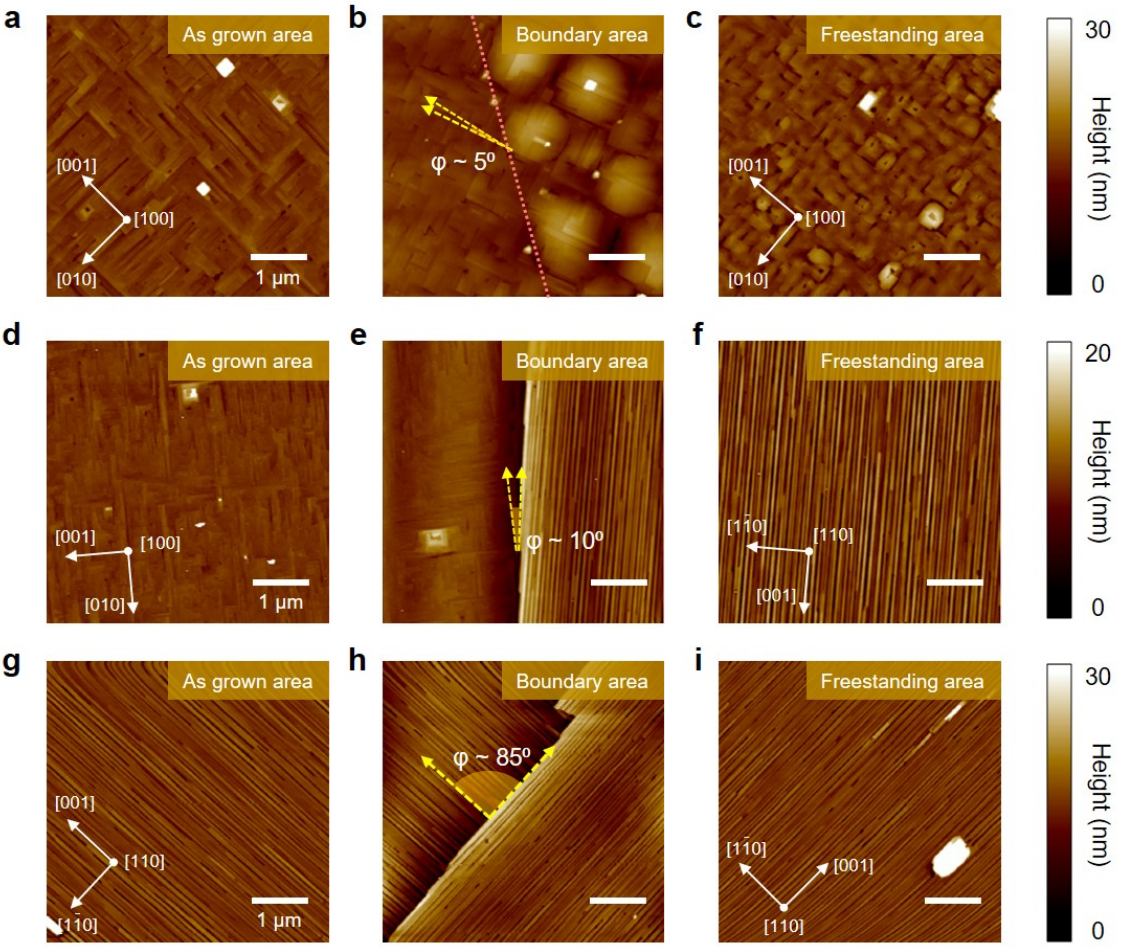

Supplementary Figure 10. BFO lateral homostructures with conjunction diversity along the out-of-plane direction. **a**, **b** and **c** BFO on (100) STO substrate and (100) FS-STO. **d**, **e** and **f** BFO on (100) STO substrate and (110) FS-STO. **g**, **h** and **i** BFO on (110) STO substrate and (110) FS-STO. The scale bars refer to 1 μm.

Supplementary References

1. Cherifi-Hertel, S. *et al.* Non-Ising and chiral ferroelectric domain walls revealed by nonlinear optical microscopy. *Nat. Commun.* **8**, 15768 (2017).
2. Xie, Y. *et al.* Artificial second-order nonlinear optics in a centrosymmetric optical material $BiVO_4$: Breaking the prerequisite for nonlinear optical materials. *ACS Omega* **4**, 1045-1052 (2019).
